# Positive Darwinian Selection in the Piston That Powers Proton Pumps in Complex I of the Mitochondria of Pacific Salmon

**DOI:** 10.1371/journal.pone.0024127

**Published:** 2011-09-28

**Authors:** Michael R. Garvin, Joseph P. Bielawski, Anthony J. Gharrett

**Affiliations:** 1 Fisheries Division, School of Fisheries and Ocean Sciences, University of Alaska Fairbanks, Juneau, Alaska, United States of America; 2 Department of Biology, Dalhousie University, Halifax, Nova Scotia, Canada; 3 Department of Mathematics & Statistics, Dalhousie University, Halifax, Nova Scotia, Canada; Instituto de Química - Universidade de São Paulo, Brazil

## Abstract

The mechanism of oxidative phosphorylation is well understood, but evolution of the proteins involved is not. We combined phylogenetic, genomic, and structural biology analyses to examine the evolution of twelve mitochondrial encoded proteins of closely related, yet phenotypically diverse, Pacific salmon. Two separate analyses identified the same seven positively selected sites in ND5. A strong signal was also detected at three sites of ND2. An energetic coupling analysis revealed several structures in the ND5 protein that may have co-evolved with the selected sites. These data implicate Complex I, specifically the piston arm of ND5 where it connects the proton pumps, as important in the evolution of Pacific salmon. Lastly, the lineage to Chinook experienced rapid evolution at the piston arm.

## Introduction

### The mitochondrion – aerobic respiration

Mitochondria house the oxidative phosphorylation system, which generates ATP, “the molecular unit of currency" of intracellular energy transfer [1]. In animals, five major protein complexes are involved in oxidative phosphorylation [2], some of which are encoded by the mitochondrial genome, which in vertebrates is circular, intronless, and encodes 22 tRNAs, 2 ribosomal RNAs, and 13 structural proteins. Complex II includes only proteins encoded by nuclear genes, whereas complexes I, III, IV, and V include both nuclear- and mitochondrial-encoded proteins [2]. In addition, the nucleotide substitution rate in mitochondrial genes is often faster than rates of many nuclear genes (Moritz et al., 1987). The rate difference was previously thought to be due to the lack a proof reading activity by the mitochondrial DNA polymerase Polγ, but recent work suggests the replication machinery does indeed proof read [3], [4]. A current theory posits that free radicals or reactive oxygen species, which are a byproduct of superoxide production by complexes of the oxidative phosphorylation system [5], damage the mitochondrial DNA (mtDNA) [6], [7] and produce a higher mutation rate.

Numerous studies have looked for evidence of natural selection on the mitochondrial genome for several reasons. Some human diseases result from mutations in the mitochondrial genome [8], which can have severe, negative metabolic consequences. It follows that other mutations may have beneficial effects on metabolism and thereby positively affect fitness. For example, it has been suggested that mutations in mtDNA resulted in cold adaptation in humans [9] although other results are not supportive [10], [11]. Population genetics studies often rely on the assumption that the segregating polymorphisms in the mitochondrial genome are neutral when, in fact, some of them may be under selection [12], [13], [14], but others have offered rebuttal [15], [16], [17]. And finally, given that the mutation rate of the mitochondrial genome is several fold higher than rates for many nuclear genes and that the oxidative phosphorylation system includes genes encoded by both genomes, the mitochondrial genome may be driving the evolution of the nuclear genome [18]. Most investigations that have looked for evidence of positive selection focused almost exclusively on primates [9], [11], [19], [20], [21], [22], [23] or analyzed single genes or complexes with *a priori* assumptions that those complexes or genes experienced positive selection [22], [23], [24]. However, those studies were unable to link putatively selected sites in the mitochondrial genome to specific structural components in the proteins and to demonstrate a phenotypic change in function, which was their ultimate goal and has been accomplished for other proteins (*e.g.* evolution of vision pigments [25]).

### Structures of the mitochondrial Complexes

High-resolution structures have been solved for Complex II (succinate∶ubiquinone oxidoreductase) [26], Complex III (cytochrome bc_1_ complex) [27], and Complex IV (cytochrome oxidase) [28]. A high-resolution structure is not available for Complex V (ATP synthase), but locations of many of the proteins within the complex are known and a mechanism of action has been proposed [29]. A high-resolution structure of the hydrophilic domain of Complex I (NADH∶quinone reductase) has been described [30]; but until recently, there has been little information on the lower, hydrophobic domain; and only low-resolution structures were available for the entire Complex I [31]. However, a recent model for the structure of Complex I, although not as well resolved as those of the other complexes, revealed a unique “piston arm" in the NuoL subunit (a homolog of ND5) in *E. coli* and provided an explanation of the proton pumping mechanism and its relationship to other protein subunits within the complex [32], [33]. Bacterial Complex I is smaller than mitochondrial Complex I; but it shares the same cofactors, performs the same function, and is highly conserved among species, which makes it a ‘minimal’ model of the mitochondrial complex [32].

### Identification of sites under positive Darwinian selection

Several methods have been used to detect the signatures of selection within gene sequences. A common method tests the null hypothesis that the ratio of intraspecies polymorphism and interspecies substitution would be the same [34]. These data are unavailable for most organisms, especially for the full mitochondrial genome. Other methods attempt to identify positive selection with an estimate of the non-synonymous/synonymous substitution ratio (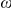) because regions of DNA are considered neutral if 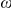 = 1, under purifying selection if 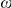
<1, and under positive selection if 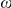
>1. Past methods have relied on averaging 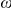 over entire genes; but sites in coding regions are usually under strong purifying (negative) selection, which would mask most signatures of positive selection that have acted upon relatively few sites.

The program PAML4 (Phylogenetic Analysis by Maximum Likelihood) [35] uses a Markov-chain model of codon substitution to identify specific sites in DNA sequences that have been under positive selection from species in a closely related phylogeny. The analytical framework permitted by the M-series models [36] implemented in PAML is advantageous for several reasons: one can analyze several aligned nucleic acid sequences simultaneously and model several classes of sites that experienced different selective pressures (different 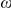) rather than averaging selective pressures over all sites; one can detect positive selection on single branches of a phylogenetic tree; and, unlike other methods that simply reject the neutral theory of evolution, one can calculate a Bayesian posterior probability that an individual codon site has been subjected to positive selection during evolution. Computation of the transition probabilities under the model also yields a formal correction for multiple hits at a site, whereas *ad hoc* methods must remove third codon positions from the analysis because of saturation (*e.g.* da Fonseca et al., 2007) [37]. Drawbacks to the M-series codon models are that they do not take into account the physicochemical change of the amino acid substitution (*i.e.* a conservative physicochemical non-synonymous substitution and a radical physicochemical non-synonymous substitution can be given equal weight) and multiple substitutions within a codon are assumed to represent several single substitution events (*i.e.* simultaneous substitutions within a codon are not allowed).

Other methods have been developed to incorporate physicochemical information into a codon substitution analysis; and although these models have been shown to fit the data better than the M-series codon-substitution methods [38], [39], the 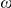 value estimated with these models now reflects the non-synonymous/synonymous bias in the protein dataset from which the significance of the physicochemical changes were drawn. Therefore 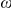 estimated from such codon models no longer has a straightforward interpretation with respect to the strength and direction of natural selection pressure. The software program TreeSAAP (Selection on Amino Acid Properties using Phylogenetic trees) [40] estimates the significance of an amino acid substitution based on the physicochemical properties and the assumption of random replacement under a neutral model of evolution. This method was shown to detect selection in highly conserved proteins [41]. A comparison of the results of these two different analyses on the same dataset can be informative because the two methods emphasize different properties of sequence evolution.

### Statistical coupling of amino acid distributions as a measure of evolutionary dependence among sites

Statistical coupling analysis (SCA) has been used to map energetic interactions among amino acid residues in protein structures [42], [43]. The idea is that co-evolution of two sites, under functional or structural constraints, leads to dependencies between the amino acid distributions at those sites, and this can be detected as thermodynamic coupling of those sites. The coupling of the two sites is quantified by an energetic coupling scalar denoted as 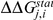. This statistic measures the change in the amino acid distribution at site *j* of a given multiple sequence alignment (MSA) that results when the data are permuted based on the amino acid distribution at another site *i*. We used a SCA here to investigate the potential for interactions between those sites identified as evolutionarily significant (via codon or TreeSAAP based analyses) and other sites having known functional significance in the ND5 protein and Complex I.

### Salmonid mitochondrial evolution

Pacific salmon (genus *Oncorhynchus*), include multiple, recently diverged species that demonstrate diverse phenotypes [44], [45], [46], [47], [48], which makes them useful organisms to address many evolutionary questions. The mitochondrial activity of one of these species has been shown to be influenced by environmental conditions [49]; and, therefore, it is likely that other species may also be. For example, differences in temperature-related respiration rates were detected between a normal, wild strain of brook char (*Salvelinus fontinalis*) and another brook char strain into which the mitochondrial genome of arctic char (*S. alpinus*) had introgressed [50]. It has been proposed that changes in mitochondrial proteins, either through selection of beneficial mutations in the coding sequences or, in the case of *Salvelinus*, through capture of an entire mitochondrial genome, represent evolutionary adaptations of respiration to different environmental conditions [51].

The recent publication of the entire mtDNA sequence for chum salmon (*Oncorhynchus keta*) [52] completed the set of mitochondrial genomes for commonly observed Pacific salmon species. Consequently, full mitochondrial genomes were available for eight species within the genus. In addition, structures are known, most at high resolution, for most of the mitochondrial proteins [26], [27], [28], [30], [53]. We examined the codon sites of 12 of the 13 protein-coding genes of the mitochondrion to identify (1) what role natural selection may have played in divergence of the mitochondrial-encoded proteins among salmonids; (2) what changes, if any, correlate with specific structures within the mitochondrial complexes; and (3) the locations of positively selected sites, if any, and whether they might have altered function of the affected protein, or reside in functionally important regions.

## Materials and Methods

### DNA Sequences

We concatenated amino acid sequences of 12 of the protein-coding genes of the mitochondrial genome for 8 species of *Oncorhynchus* [36], [54]. The 12 genes were: ND1, ND2, ND3, ND4, ND4L, ND5, COX1, COX2, COX3, ATP6, ATP8, and Cytochrome b. The ND6 gene was excluded because it is encoded on the opposite strand. Sequences that were aligned were GeneBank accessions for *O. tshawytscha*, #AF392054.1; *O. kisutch*, #EF126369.1; *O. nerka*, #EF055889.1; *O. masou*, #EF105342.1; *O. mykiss*, #DQ288271.1; *O. keta*, #AP010773; *O. clarki*, #AY886762.1; and *O. gorbuscha*, #EF455489.1. A second larger set of sequences was analyzed that also included full mitochondrial genomes from *Salmo salar*, AF133701.1; *Salmo trutta*, AM910409.1; *Salvelinus fontinalis*, AF154850.1; *Salvelinus alpinus*, AF154851.1; *Thymallus arcticus*, FJ872559.1); *Thymallus thymallus*, FJ853655.1; and *Coregonus lavaretus*, AB034824.1. Sequences for the NuoL gene from *E. coli* and the Nqo12 gene from *T. thermophilus* were taken from Efremov *et al.* 2010.

Sequences for the MSA used in the statistical coupling analysis were collected as follows: The NCBI database was searched for all ND5 sequences for ray-finned fishes with CLC Bio (Denmark). This generated 931 sequences for the initial MSA, which were assembled by using the progressive alignment algorithm [55] applied in CLC Bio (Denmark). The ND5 sequence has undergone the most significant structural changes in evolution as compared to the other mitochondrial encoded proteins, specifically in the C-terminal portion of the protein [31]; and this has led to a MSA that contains many gaps. A high-resolution structure is not yet available that is sufficient for a structural-based alignment. Therefore, we used the 15 sequences from Salmonidae as a core set of sequences; and then purged all sequences with large regions of ‘gaps’. This resulted in 428 sequences for our MSA that were used for the SCA (below). The SCA also requires a reference set of sequences for estimating an appropriate null amino acid frequency distribution. In this case, we compiled the full set of 925,462 mitochondrially-encoded amino acid sequences of metazoans available from GenBank in April 2011. Secondary structure prediction was done with TMHMM v2.0 [56]) for the core set of 15 sequences from Salmonidae.

### Inference of phylogenetic trees

We used MrBayes 3.1 [57] to construct trees from the concatenated sequence for 12 genes of the mtDNA genome for eight species of *Oncorhynchus* and also for the larger data set. The ‘nucleotide model’ parameter was set to ‘codons’, rather than ‘nucleotides’, the MCMC chain was run for 200,000 generations; and 2,000 samples were taken to estimate the posterior distribution. Two criteria were used to test convergence of the chains: (1) the standard deviation of split frequencies was less than 0.01, and (2) the potential scale reduction factors (PSRF) [58] were close to 1.0 for all parameters.

### DNA sequence analysis

We used the CODEML program in the PAML4 software package to conduct a random-sites analysis [35]. The random-sites analysis is based on a codon model that explains the data as a mixture of several categories of evolution, each characterized by an independent parameter for selection pressure (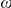). In these models the substitution rate from codon *i* to codon *j* within a specific category of the model is specified as,
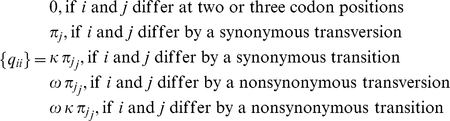
The transition probability matrix is calculated as 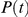 = 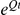, where 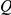 = 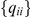
with the standard theory. Evolution is assumed to be independent among sites, so the likelihood of the data is computed for each codon position in the alignment and the log of those site-likelihood scores are summed to generate a log-likelihood score for the entire dataset. The likelihood function is maximized with respect to the values of the parameters 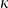, 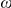, and *t* (the branch length, which is the expected number of nucleotide substitutions per codon averaged over all sites). The parameter 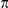 is obtained from the observed nucleotide frequencies.

We applied the recommended subset of six M-series models to investigate variation in selection pressure among sites [59]. These models are M0 (one ratio), M1a (nearly neutral), M2a (positive selection), M3 (discrete), M7 (beta), and M8 (beta & 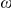). Parameters were estimated for each model by maximizing the likelihood of the data, and the significance of parameters was tested by contrasts of models that included a parameter of interest (*i.e.* an alternative model) with the model that did not include it (the corresponding null model). The contrasts between models were tested with a log-likelihood ratio test (LRT) to determine if the alternative model was a significant improvement over the corresponding null model. The simplest model, M0, assumes that all sites belong to a single “class", and 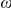 is estimated from the data. Model M1a has two classes of sites; one class is for neutral sites (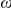 fixed at 1) and another for purifying selection (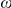 constrained <1). Model M2a extends M1a with a third class of positively selected sites (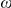
>1). The M1a–M2a LRT is an explicit test for the presence of positively selected sites. Model M3 (the discrete model) extends M0 to include three classes of sites, and the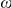 for each class of sites is estimated independently from the data. A LRT of M0 against M3 is a formal test for variable selection pressure among sites. Model M7 models variable selection pressure under the constraint that 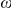 is between 0 and 1 with a beta distribution (with shape parameters *p* and *q*). M8 extends M7 with a class of positively selected sites (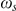
>1). The M7–M8 LRT also is an explicit test for the presence of positively selected sites.

We wished to identify which specific sites in the coding sequences experienced positive Darwinian selection. Therefore, we estimated both a Naïve Empirical Bayesian (NEB) and Bayes Empirical Bayesian (BEB) posterior probability (*PP*) for the sites that experienced positive selection for M2a, and M8, and a NEB probability for M3. The NEB method assumes that all parameters are estimated without error whereas the BEB incorporates a prior distribution that accounts for errors in parameter estimates. The data at a codon site was considered consistent with having evolved by positive Darwinian selection if it had a BEB posterior probability that exceeded 90% for a model category with 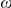
>1. Inferences based on posterior probabilities are substantially improved if they are conditioned on the results of a joint analysis of all codon sites [60]. Specifically, for a site to be considered positively selected, at least one category of a codon model must have an estimate 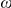 that exceeds 1; and either the LRT of M1a vs. M2a or the LRT of M7 vs. M8 must be significant. Although M0 vs. M3 is not an explicit test for selection, it does allow one of the three categories to exceed 1, which would be additional evidence of positive selection.

Random-site analyses are conservative tests because they only detect sites that have experienced positive selection if that selection has occurred at that site across multiple lineages; it can miss sites that have undergone positive selection only on one lineage. Therefore, we performed a branch-site test, which can detect if a site has undergone positive selection in only one lineage of a tree [61]. In this analysis, the branches of the phylogenetic tree are divided into foreground and background branches and selection is tested with a comparison of two models. We used the contrast of Model A in which the foreground 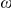 = 1 (the null) and Model A in which the foreground 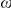
>1 (the alternative) as formulated by Zhang *et al.* 2005. In our analysis, Chinook was chosen as the foreground branch.

The full mitochondrial genome data (excluding ND6) were also analyzed with models implemented in TreeSAAP [40], which compares the observed distribution of physicochemical changes inferred from a phylogenetic tree with an expected distribution based on the assumption of completely random amino acid replacement expected under the assumption of neutrality. Statistical significance is determined based on a goodness-of-fit test under a chi-square distribution. Tests were performed for 31 different physicochemical properties with a sliding window size of 15 amino acids. Significance was scored for each site and divided into four groups (*p*>0.05, *p*<0.05, *p*<0.01, and *p*<0.001) for each of the 31 properties. In addition, each substitution was scored on a scale of 1–8, where 1 is a conservative change and 8 is a radical change that represent stabilizing and diversifying positive selection, respectively [40]. We only considered sites as significant with *p*<0.001.

### Statistical Coupling Analysis

The SCA is based on a measure of statistical energy denoted 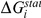. This statistic is derived from a vector of binomial probabilities of observing exactly *y* instances of amino acid *x* at site *i* in a MSA (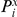). The binomial probability is computed using the empirical frequency of amino acid *x* in a reference data set as the expected probability of an individual occurrence of amino acid *x*. Here, the reference data set is the full set of 925,462 mitochondrially-encoded amino acid sequences of metazoans. These binomial frequencies are converted to the statistical energy statistic as:
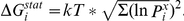
Where the term kT* is the arbitrary unit of energy described in previous work [42]. Here, we set kT* = 1/*M*, where *M* is the number of sequences in the MSA. This is a modification from earlier analyses [42] for scaling alignments of different size. We also used an updated approach because the SCA methods no longer scale 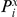 by the probability of observing amino acid *x* taken over all sites in the MSA. A low 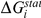 indicates that the amino acid distribution at site *i* is close to that in the reference dataset, and this can be interpreted as a signal for lack of evolutionary constraint at that site. A high 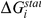 indicates a large divergence between the amino acid distributions in the reference data and site *i*, and this can be interpreted as signal for strong selective constraints operating at that site.

Statistical coupling between sites is quantified by the magnitude of the change between the vector of binomial probabilities at site *j* of a given MSA that results from a perturbation of the amino acid distribution at another site *i*. The binomial probability of amino acid *x* at site *j* after such at perturbation is denoted 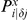. Here, we performed the perturbation at the seven different sites (*i*) that we identified as having evolved under positive selection pressure (*i.e.*, 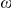
>1). The perturbation was performed by sub-sampling the MSA for sequences that have the most common amino acid at site *i*. The scalar coupling energy was computed as:
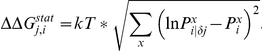



The SCA assumes that the statistical free energy (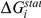) can be reliability estimated for each site in a MSA before and after perturbation of the amino acid distribution at that site. This requirement implies three key properties of the sample of sequences within the MSA, which we tested prior to carrying out the SCA. The results of the tests are summarized below.

The first assumption is that the MSA represents a sample of sequence evolution that has reached a state of statistical equilibrium. If at equilibrium we expect that sites that are subject to very weak constraints or are completely free from constraints, they should have amino acid distributions closest to the average over all the proteins in the reference set. Because we analyzed a single protein, and because we could not perform a structural based alignment, our ability to sample was restricted and our MSA does not include sites completely free from selective constraints. Hence, we focused sites subject to very weak constraints, as identified by low 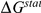 (<0.3) and compared the amino acid frequencies at such sites to those derived from the reference set. These sites are 077, 209, 506, 525, 573, 576, 578, and 609–612. The frequencies at these sites are most similar to the reference data set, although in no case is their distribution an exact match to the reference data set (Figure S1a). Sites with higher 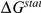 differ substantially from the reference set (Figure S1b).

The second assumption is that the MSA is large enough so that site-wise perturbation does not negatively impact estimates of the equilibrium distributions after the perturbation. If the MSA is large enough, sites with low 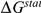 in the full MSA will have low 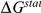 after random removal of sequences. To validate this expectation we carried-out ten replications where we randomly deleted half of the sequences from the MSA. Comparison of scores for the eleven low-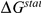 sites listed above reveals that the full MSA is robust to random elimination of sequences (Table S1). We then re-tested this expectation for the seven sites identified that were the target of the SCA because they appear to have evolved under positive selection pressure (520, 521, 525, 526, 575, 576, and 577) (see Results). In this case the size of the perturbation (*s*) was based on the most frequent amino acid at each of those sites (Table S2), which is how perturbations are carried out for the SCA. Again, the similarity of 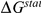 scores for the full MSA and ten random samples of size *s* (Table S2) suggest that the sizes of the sub-alignments were sufficient for the SCA.

The third assumption is that perturbation of the full MSA does not, by chance, yield a sample dominated by recent evolutionary divergences, thereby inflating the level of non-independence among sequences. The standard deviations of the 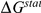 scores for the random elimination carried out above are small (Table S1), which suggests that phylogenetic effects might have only a minor impact. However, to further investigate this we sub-sampled the full MSA to maximize phylogenetic disparity among the sequences in the subsample. We sampled the full MSA under the constraint that all sequences must have >5% (uncorrected) sequence dissimilarity. The removal of all of the closely related sequences (<5%) yielded a MSA of 241 sequences. This represents a 44% reduction in size, indicating that the full MSA includes many recent evolutionary origins. Thus, the full MSA, as well as the typical random sample, has a different phylogenetic distribution than the 241 sequences sampled to maximize evolutionary disparity. 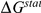 scores for the latter MSA are close to 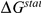 scores for the full MSA (Table S1). However, there is a tendency for 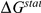 to be slightly higher than in the full MSA, which suggests the possibility that phylogenetic relatedness of sequences sampled from the full MSA could influence 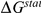 scores, and results should be interpreted accordingly.

### Generation of 3-dimensional structure

The 3-dimensional structure was drawn with Jmol (Jmol: an open-source Java viewer for chemical structures in 3D. http://www.jmol.org/) with the file PDB ID3M9S. Contrasting colors were used to highlight domains. Some proteins appear as fragments due to the low resolution of the crystal structure (Efremov et al., 2010).

## Results

### Phylogenetic trees

**Figure 1:**
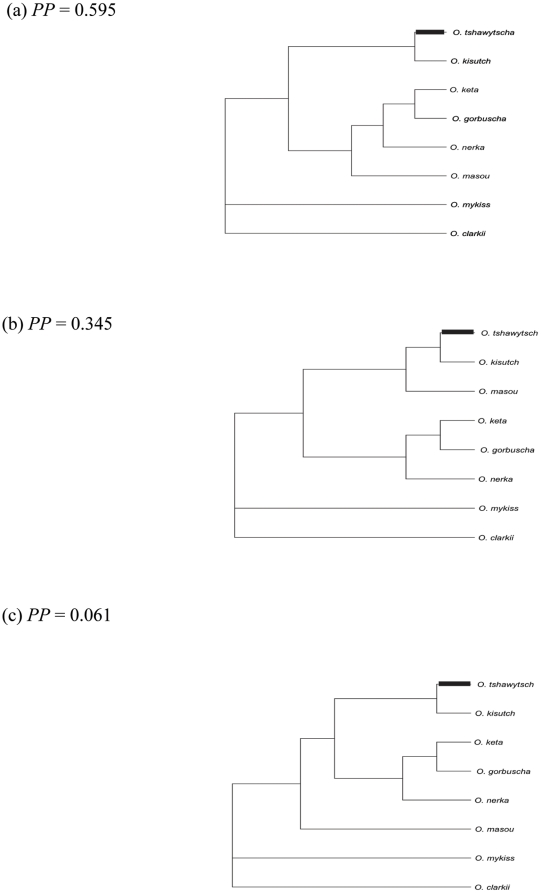
Phylogenetic Trees inferred using MrBayes. Phylogenetic trees estimated from the mtDNA coding sequence data from eight species of Pacific salmon. The Bayesian analysis predicted three trees with posterior probabilities of (a) 0.595, (b) 0.343, and (c) 0.061. The foreground branch for the branch-site analysis is identified with a bold line. Trees were drawn without distances because Mr. Bayes estimates distances for only the most probable tree.

The three trees that were inferred from the analysis of eight species of *Oncorhynchus* had posterior probabilities of 0.595, 0.343, and 0.061 (Figure 1). Three trees were also inferred from the larger data set that included an additional seven sequences, which represented more divergent salmonids (see DNA Sequences) and for which posterior probabilities were 0.909, 0.084, and 0.007. The tree for the eight species of *Oncorhynchus* varied in the placement of *O. masou*. In the most probable tree for that analysis, *O. masou* was most closely related to the group represented by *O. keta*, *O. gorbuscha*, and *O. nerka*. The second most probable tree placed *O. masou* with the *O. kisutch* and *O. tshawytscha* group (Figure 1). All three trees inferred from the 15 species in Salmonidae placed *O. masou* with the *O. gorbuscha*, *O. keta*, and *O. nerka* group (Figure S2).

### Overview of detection of positively selected sites

We conducted an amino acid site-based analysis in several steps. First we tested for a signal of positive Darwinian selection within the set of salmonid mitochondrial coding sequences (except for ND6) with a random-sites analysis with models implemented in PAML4. These models can detect long-term positive selection if non-synonymous changes occur within a codon site in multiple taxa (note the unit of interest here is the codon and not the amino acid). Next, we carried out a branch-site test with the same set of data. Branch-site tests can reveal evidence for positive selection on one lineage of closely related taxa; the nonsynonymous changes do not need to occur in several taxa, only in one or a few. Third, we carried out a robustness analysis to determine the strength of our results. Fourth, we carried out an analysis in the program TreeSAAP to determine if selection played a role to set the physicochemical constraints on the same amino acid sites that were identified with the random-sites analysis (note the unit of interest in this analysis is the amino acid and not the codon). Finally, we superimposed the positively selected sites that were identified by PAML4 and TreeSAAP onto the 3-dimensional structure of Complex I to determine if the selected sites correlated with functionally important areas of the proteins (*e.g*. ubiquinone binding pocket, iron-sulfur clusters, etc) and then carried out a SCA to search for evidence that other sites within the ND5 protein may have co-evolved with the selected sites.

### Random-sites analysis

We fit several Markov models of codon evolution (Table 1) to the concatenated set of gene sequences by using maximum likelihood, which allows estimation of model parameters and tests of hypotheses about the distribution of selection pressures. The data provided a very strong signal for variable selection pressure among sites (Table 2). The M0-M3 LRT was highly significant (*P*<10^−3^), and parameter estimates suggested very different levels of selection pressure (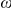) for different classes of sites (Table 3). These data also produced a signal for a small fraction of sites that had evolved under positive Darwinian selection pressure because two models that permit such evolution (M3 and M8) had a class of sites with an estimated value of 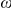 much larger than 1. However, parameter estimates under M2a indicated an absence of positively selected sites. The formal statistical tests of this hypothesis follow this disagreement: LRT of M1a-M2a was not significant (*p*>0.999) whereas the LRT of M7-M8 was (*p*<0.004). These results mirror the known statistical properties of these models and tests [62]. Specifically, the M1a-M2a LRT is a much more conservative test because it has lower power than the M7-M8 LRT when positively selected sites truly exist in the data. Concordance between two models with very different formulations (M3 and M8) corroborates the significant M7-M8 LRT result.

**Table 1:** Site-models used in to analyze Pacific salmonid mitochondrial gene sequences, the numbers of parameters estimated for each model (p), and all of the parameters included in each model.

Model	p	Parameters
M0 (one ratio)	1	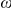
M1a (neutral)	2	*p*_0_ (*p*_1_ = 1−*p*_0_) 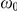 <1, 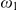 = 1
M2a (selection)	4	*p*_0_, *p*_1_ (*p*_2_ = 1−*p*_0_−*p*_1_) 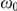 <1, 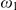 = 1, 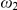 >1
M3 (discrete)	5	*p*_0_, *p*_1_ (*p*_2_ = 1−*p*_0_−*p*_1_) 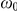 , 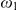 , 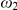
M7 (beta)	2	*p*, *q*
M8 (beta & 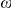 )	4	*p*_0_ (*p*_1_ = 1−*p*_0_), 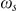 >1; *p*, *q*

Descriptions of each model are given in the Methods section.

**Table 2:** Model comparison via log-likelihood ratio tests.

Null Model	Experimental Model	Probability	Testing for	Result
Model 0 (one ratio)	Model 1a (nearly neutral)	<10^−3^	Do 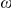 s differ among sites?	Yes
Model 1a (nearly neutral)	Model 2a (positive selection)	>0.999	Is there positive selection?	No
Model 0 (one ratio)	Model 3 (discrete)	<10^−3^	Do 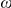 s differ among sites?	Yes
Model 7 (beta)	Model 8 (beta & 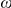 )	0.004	Is there positive selection?	Yes

**Table 3:** Site-models, their log likelihood values, and parameter estimates for Pacific salmonid mitochondrial gene sequences.

Model Number	Log Likelihood	*k*	Parameter Estimates
M0 (one ratio)	−29512.600	6.31	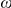 = 0.02393
M1a (nearly neutral)	−29395.916	6.37	*p*_0_ = 0.98039 , *p*_1_ = 0.01961, 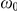 = 0.01088, 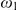 = 1
M2a (positive Selection)	−29395.917	6.37	*p*_0_ = 0.98039, *p*_1_ = 0.01961, *p*_2_ = 0, 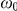 = 0.01088, 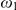 = 1, 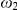 = 92.78523
M3 (discrete)	−29383.608	6.33	*p*_0_ = 0.94145, *p*_1_ = 0.05791,*p*_2_ = 0.00063, 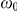 = 0.00429, 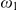 = 0.34495, 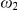 = 3.52065
M7 (beta)*	−29390.205	6.32	*p* = 0.01284, *q* = 0.29767
M8 (beta & 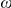 )*	−29384.575	6.33	*p*_0_ = 0.99928, *p*_1_ = 0.00072, 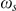 = 3.29192, *p* = 0.01348, *q* = 0.26069

* The p and q in these models represent parameter estimates for the beta distribution.

### Branch-site analysis

The random-sites analyses described above have low power to detect episodes of positive selection when positive selection acts only at certain sites over just one or a few lineages in the tree [63]. Therefore, we performed a “branch-site test", as formulated in Zhang et al., (2005) [64]. The branch of interest is specified in branch-site Model A prior to the analysis and is hereafter referred to as the foreground branch. In our analysis, the foreground branch is the branch that leads to Chinook (Figure 1). We chose that branch because there is a lysine/proline substitution at position 522 near the selected sites (see below), which is biochemically noteworthy because proline can induce bends and cause changes in 3-dimensional protein structure [65]. We employed the branch-site model (called Model A), which is an extension of model M2a, because the resulting LRT is more conservative than tests based on branch-site Model B. The important feature is that the null Model A only permits purifying (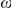
<1) or neutral evolution (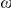 = 1) in the foreground branch, and the alternative Model A allows values of 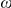 to exceed 1 (positive selection) in the foreground branch. Parameter estimates under the alternative model indicated that 97.9% of the sites are under purifying selection in both the foreground and background branches, 1.63% of the sites are neutral in both foreground and background branches, and 0.40% have been subject to an episode of positive selection in the foreground branch (Table 4). The LRT of the null and alternative models are highly significant (*p*<10^−3^) and substantiate the signal for positive selection.

**Table 4:** Null and alternative formulations of Branch-site Model A and their parameter estimates for Pacific salmonid mitochondrial sequences.

Model	Site Class	0	1	2a	2b
Model A (Null)	proportion	0.955	0.016	0.029	<10^−3^
	Background 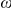	0.010	1.000	0.010	1.000
	foreground 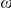	0.010	1.000	1.000	1.000
Model A (Alternative)	proportion	0.979	0.016	0.004	<10^−3^
	background 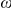	0.011	1.000	0.011	1.000
	foreground 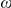	0.011	1.000	225.5	225.5

### Robustness analyses

We conducted additional analyses to evaluate the robustness of signal for positive selection in these data. The instantaneous rate change of codon *i* to *j* depends on the parameter 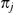 (equilibrium frequency of codon *j*), and 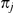 can be estimated in several ways. For most analyses we estimated the 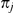
's from the nucleotide frequencies observed at each of the three codon positions separately (F3×4). The parameter, 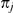 *_j_*, can also be estimated from the nucleotide frequencies observed in the entire data set (F1×4) or under the assumption that all nucleotide substitutions are equivalent (Fequal). When we reran both the M7–M8 LRT and Model A-based LRT under alternatives Fequal and F1×4, the same sites were identified as under positive selection with nearly the same posterior probabilities that were observed under F3×4. Next, we added mtDNA genomes of seven more distantly related salmonid species to the analysis (Fig. S2). Again, the LRTs based on the M7–M8 model pair, and restricted and unrestricted versions of Model A, were significant. Recall that three trees were predicted with MrBayes. The tree with the highest posterior probability (0.595) was used to obtain the results presented above. To assess the sensitivity of these results to tree topology we repeated our analyses with the remaining two tree topologies (*PP* = 0.343, and *PP* = 0.061). Results were not sensitive to topology; the LRTs based on the M7–M8 and Model A were significant and the same sites were identified as having experienced positive selection with similar posterior probabilities. In sum, these two statistical tests for positive selection and empirical Bayes site identification under the alternative models (M8 and Model A with 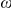
>1) were robust to the formulation of parameter *p*, additional taxon sampling, and tree topology. It is unlikely that they represent type I errors.

### Bayesian probabilities for sites under positive selection

To relate the action of positive selection to the 3-dimensional structure of Complex I, we obtained site-specific information about the strength and direction of natural selection pressure on a site-by-site basis. Individual sites were inferred to have evolved under positive selection if they had a BEB posterior probability >0.90 and belonged to a model category with 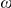
>1 (Table 5). These analyses revealed sites subjected to two different forms of positive selection: long-term and episodic. A site is characterized as long-term when it is subject to positive selection in most or all branches of the phylogeny. Models M3 and M8 are formulated to detect long-term selection, and two sites in the ND5 gene had high probabilities (>0.90) of positive selection under both of those models. The values given for the site models in Table 5 are for Model 8. A different set of sites was identified as subject to episodic selection; positive selection was detected at five sites in the lineage that led to *O. tshawytscha*. These results are noteworthy, because all seven sites from the 12 structural gene alignment are located in a small region of the ND5 gene. These seven sites (long-term and episodic) were identified with both the NEB and BEB methods.

**Table 5:** Bayesian posterior probabilities of sites under selection in *Oncorhynchus* spp. Branch-Site analyses revealed episodic and Site-Model analyses long-term positive selection.

Gene Name	Amino Acid Position	NEB	BEB	Type positive selection
ND5	520	1.000	0.937	Episodic
ND5	521	1.000	0.950	Episodic
ND5	525	0.968	0.980	Long-term
ND5	526	0.971	0.983	Long-term
ND5	575	0.993	0.928	Episodic
ND5	576	0.997	0.907	Episodic
ND5	577	0.997	0.906	Episodic

### TreeSAAP Analysis

**Figure 2:**
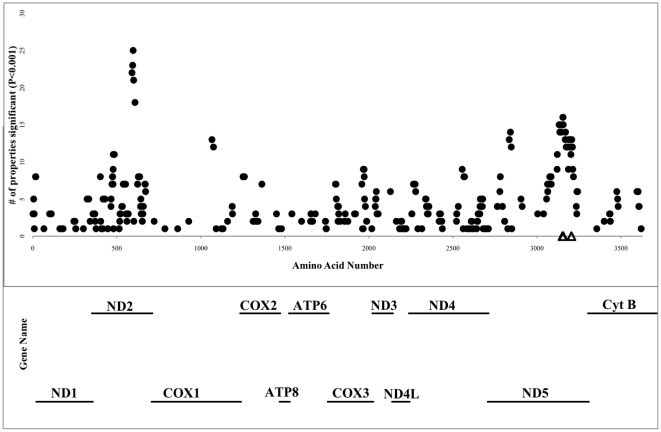
Summary of selection on the mitogenome of *Oncorhynchus*. The 12 mitochondrial coding genes are represented and labeled (the figure does not include tRNAs or the ND6 gene). Filled circles represent positively selected sites (*p*<0.001) by TreeSAAP analysis. The dataset was analyzed for significance 31 times (for each of the 31 physicochemical properties). The y-axis represents the number of properties for which that site was determined to be subject to natural selection. Open triangles are positively selected sites identified by PAML analysis.

The program TreeSAAP scored sites as having been subject to natural selection with respect to 31 different physicochemical properties according to the change in the physicochemical property of each amino acid substitution. The program indicated that some sites were selected as a consequence of more than one property (Figure 2), and that some of those changes were radical changes. These results corroborate the results obtained under the codon models because there is a very strong signal for selection on 15 different physicochemical properties at each of the sites detected in ND5 with the codon and amino acid models. In addition, three sites in the ND2 gene also demonstrated a strong signal for positive Darwinian selection for more than 20 properties, half of which were radical changes. Finally, the TreeSAAP analysis revealed very strong purifying selection on the genes for ND1, the COX genes, ATP6, and ATP8.

### Structural location of positively selected sites

**Figure 3:**
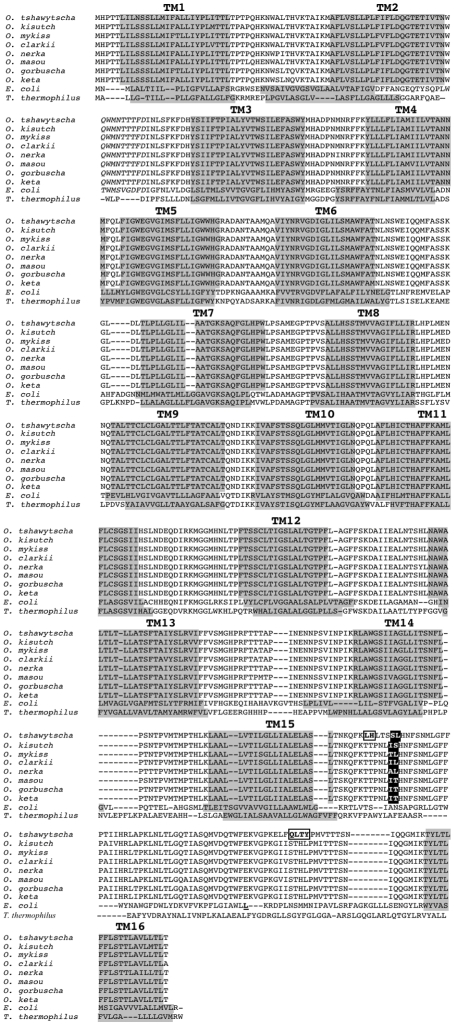
Sequence alignment and secondary structure prediction of the ND5 gene for eight species of *Oncorhynchus*, the NuoL gene for *E. coli* and the Nqo12 gene from *T. thermophilus*. The amino acids in white bold and black background are predicted to be under long-term positive selection in eight species of *Oncorhynchus* (long-term selection). The amino acids in boxes were determined to have been under positive selection only in *O. tshawytscha* (episodic selection). Transmembrane domains are shown in bold lettering above gray background-colored amino acids.

**Figure 4:**
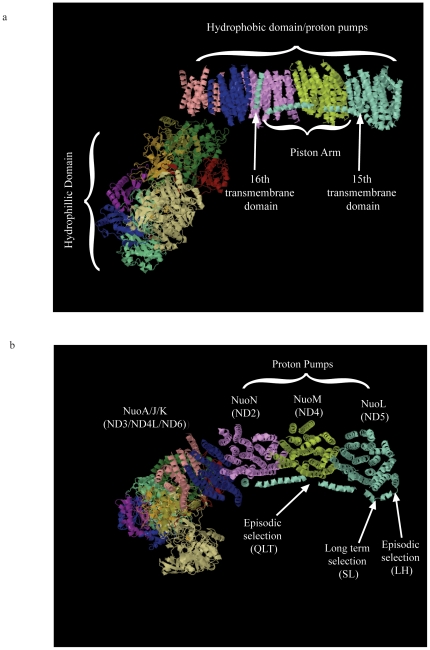
3-dimensional crystal structure of Complex I *from E. coli*. (a) Major domains of the complex. The hydrophobic domain contains the proton pumps and the recently discovered piston arm is identified. The 15^th^ and 16^th^ transmembrane domains that anchor the piston arm are also shown. (b) Individual proton pumps are labeled. Arrows point to the likely locations of sites under both episodic- and long-term selection and the amino acids from the *O. tshawytscha* sequence are given below each arrow (see Results).

How do these observations relate to the structure and function of Complex I? The recently described [32], [33] 3-dimensional structure of Complex I in *E. coli* revealed a novel “piston" arm that is part of the NuoL subunit (a homolog of ND5) and likely connects the three proton pumps: NuoL (ND5), NuoM (ND4), and NuoN (ND2). The proposed structure provides a mechanism in which transfer of electrons from NADH to ubiquinone by the upper hydrophilic arm of Complex I induces a conformational change in the lower hydrophobic region. The linking arm from NuoL then causes a coordinated shift of all three proton pumps (NuoL, NuoM, and NuoN) to move H^+^ ions across the inner membrane, which creates the electrochemical gradient and drives the formation of ATP by Complex V in the oxidative phosphorylation system. A multiple sequence alignment in conjunction with the prediction of transmembrane helices (Figure 3) allowed us to identify the locations of the positively selected substitutions in Complex I for the ND5 protein. Our codon-based analysis predicted that seven sites experienced positive selection (two long-term and five episodic) and all reside in the piston arm of ND5. Four of those sites, two long-term (#525 and #526) and two episodic (#520 and #521), are located at the origin of the piston arm as it emerges from transmembrane helix 15 in ND5 (Figure 4), and likely interact with the ND5 proton pump itself. The remaining three sites (# 576, #577, and #578), all episodic, correspond to the residues in the “piston" arm that may interact with the proton pump of the adjacent protein ND4, Figure 4).

The TreeSAAP analysis confirmed a strong signal for positive selection on the same amino acids in the piston arm of ND5, and also at three amino acids in one of the other pumps, ND2 (Figure 2). Although the PAML analysis did not identify the three sites in the ND2 gene as positively selected, the TreeSAAP results indicate a signature of diversifying selection at the level of amino acid physiochemical property because nearly half of the substitutions by property analysis were radical (10 of 21 properties were identified as a category 7 or 8 change). One of the sites in the ND2 protein resides in a hydrophilic loop, and the other two reside adjacent to the first site in the eighth transmembrane helix as predicted by TMHMM v2.0 [56] (data not shown). Although the sequence alignment of ND5 (Figure 3) and ND2 (data not shown) shows that the transmembrane domains tend to be highly conserved, a previous study identified a positively selected site also in the eighth transmembrane domain of ND2 [37], another study predicted selection of a site in the sixth transmembrane domain of ND2 [24], and third study identified selection in the transmembrane domains when compared to surface codons, but did not identify specific sites [23].

### Statistical Coupling Analysis

**Figure 5:**
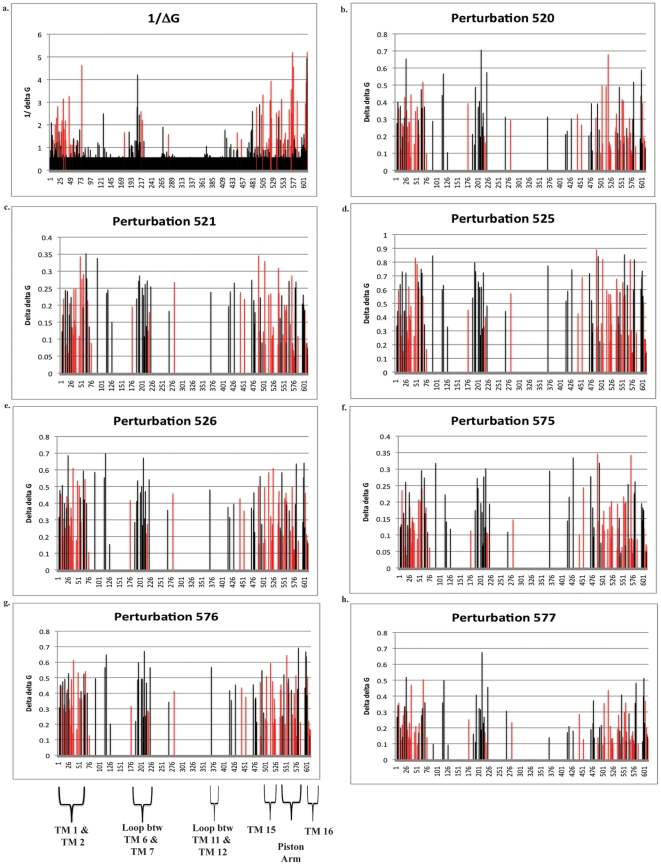
**Results of the statistical coupling analysis from 428 ND5 amino acid sequences.** The graph of the inverse 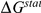 (1/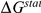) shows sites that are ‘unconstrained’ (have a low 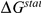 value or high 1/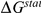 value) in the ND5 protein compared to a random distribution of amino acids at each site in the MSA (5a). The inverse of 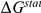 is shown for visual simplicity. The y-axis is the 1/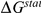 and the x-axis is the amino acid position. These could be ‘neutral’ sites or those under positive diversifying selection. The remaining graphs (5b–5h) are the 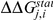 values for each site in the MSA after perturbation for each of the selected sites. Red bars are those sites that are considered significant by TreeSAAP analysis in 15 species of Salmonidae only.

Many of the 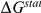 values for the full MSA were high, which indicates a highly constrained amino acid distribution at those sites (Figure 5a). Such a result is expected for sites dominated by strong purifying selection where the distribution of selective coefficients has been relatively constant over time. More over, we observed a correlation between 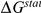 and posterior mean *ω* at a site (data not shown), which is consistent with the above interpretation. However, our analysis of this dataset also reveals that inclusion of closely-related lineages has a small, but systematic, influence on 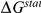 (Table S1). The problem arises because similarities in state at a given site will be due to both the effect of purifying selection pressure and inheritance from common ancestor; thus, 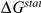 is also influenced by the degree to which closely related sequences have been sampled. Because this problem will be most severe at the strongly conserved sites, and because ∼70% of the sites were nearly fixed for one amino acid, we chose to restrict subsequent analyses of statistical coupling (via 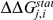) to just the unconstrained sites (*i.e.* those having moderate to low 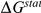 scores). Furthermore, we graph 1/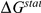 to facilitate visualization of such ‘unconstrained’ sites.

Although we have determined that the data are generally suitable for the SCA, we are still faced with the task of inferring if the amino acid distributions at two specific sites are indeed dependent on each other. Estimating 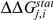 only for those sites with moderate to low *DG* does not address this issue. A randomization-based method has been proposed for testing specific estimates of 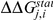 [42]; however, we observed that *p*-values derived from their method are inappropriate because they do not actually test the null case that 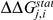 = 0 (data not shown). We believe that a test based on the non-parametric bootstrap [66] would be appropriate for highly divergent sequences. Alternatively, a parametric bootstrap [67] would be more appropriate for cases where 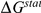 is substantially impacted by phylogenetic non-independence among sequences. Development and testing of such hypothesis-testing frameworks is beyond the scope of this paper, and will the subject of future work.

Rather than forming the basis of a hypothesis test, we employ 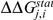 as an objective means of exploring the signal in the data and generating novel hypotheses. Statistic-based data exploration is a widely accepted alternative to formal hypothesis testing [59]; indeed, we already did this to classify sites in the MSA according to its Bayesian posterior probability of having 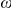
>1. For the SCA, we rank ordered the 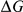 values for all variable sites and selected the lowest 50% for further analysis. We then measured the statistical coupling of those sites (via 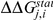) to each of the seven sites we previously classified as having evolved under positive selection pressure (520, 521, 525, 526, 575, 576, and 577). We present separate plots of the statistical coupling of each of those seven sites (Figure 5b–h). All the plots show coupling with the same four regions of the ND5 protein: (1) the first and second transmembrane domains, (2) the hydrophilic loop between the 6^th^ and 7^th^ transmembrane domains, (3) the hydrophilic loop between the 11^th^ and 12^th^ transmembrane domains, and (4) the piston arm motif, which includes both the 15^th^ and 16^th^ transmembrane domains (Figure 4). In addition, many of these same sites were positive by TreeSAAP analysis (Figure 5b–5h, red bars).

### Addendum to manuscript

As our manuscript was going to press, a higher resolution structure of Complex I was published [68]. In that work, the positions of the transmembrane domains of each of the proton pumps, NuoL (homolog of ND5), NuoM (homolog of ND4), and NuoN (homolog of ND2), in relation to the piston arm were shown and the authors proposed that hydrogen ions move across the inner-mitochondrial membrane via specific, highly conserved lysine residues in transmembrane domains 7 and 12 (TM7 and TM12) in each of the proton pumps.

These results and proposed mechanism of action are coincident with our SCA predictions. We detected co-evolution of amino acid sites near both TM7 and TM12 in the ND5 protein (Figure 5 our work) and the sites that were consistent with positive selection in Salmonidae. In addition, our TreeSAAP analysis identified three sites in the ND2 protein that likely had experienced diversifying positive selection (sites # 271, #274, and #278 in Salmonidae). A multiple sequence alignment showed that these three sites correspond to amino acids #403, #406, and #410 in NuoM, which are all in TM12, adjacent to the lysine that is proposed to transfer the hydrogen ions across the inner-membrane. It is likely that the sites in ND2 and ND5, which we showed to have experienced positive selection during the evolution of salmonids, are instrumental in the transfer of hydrogen ions across the inner mitochondrial membrane and, consequently, affect the energy status of the cells in which they reside.

## Discussion

### Overview

This is the first study that analyzed the coding sequences in the full mitochondrial genome (except ND6), presented statistically supported evidence of positive Darwinian selection at specific amino acids sites, and related those specific amino acid residues to the 3-dimensional context of the multi-subunit protein. There have been numerous other efforts to survey mitochondrial genomes for signatures of positive selection, but most were limited in the scope of possible inferences. Limitations arose either by an analytical framework that only permitted rejection of the neutral theory of evolution jointly for a complete set of mitochondrial sequences (*i.e.* tests for selection at specific sites were not possible) or they sampled only a subset of mitochondrial proteins (*e.g.* Elson et al., 2004; Nachman et al.,1996; Wise et al., 1998; Zink 2005). Only two studies attempted what we have done here. One study compiled evidence for selection in the mitochondrial genomes of primates from numerous other studies [20]. Their focus was on selection in Complexes III and IV of the mitochondrial genome, but it did not address selection in Complex I and had minimal information for Complex V. A second study employed TreeSAAP to identify positively selected sites in 41 mammalian species. As with our work, a signal for positive selection was detected in ND5, but they were unable to place the selected sites of the ND2, ND4, and ND5 proteins within a structural-context because the structure of Complex I was not known at the time [37]. Interestingly, that study and others also detected positively selected sites in the C-terminal portion of ND5 [21], [31], [37], [69]. Finally, a study of the hydrophobicity of the mitochondrial encoded ND proteins of 11 different phyla indicated rapid evolution at the in the same location [31]. This observation, when taken together with our results, suggests the possibility that the piston arm of ND5 might have been the target of adaptive modification in a wide variety of lineages. A wider investigation of piston arm evolution appears warranted.

We observed that changes in the piston arm and, consequently, proton pumping, may have influenced fitness during the evolution of Pacific salmon species. Because these species are important for economic, conservation, and recreational reasons, a plethora of data exist that can be correlated to functional studies. Behnke provides a thorough description of the phenotypes of North American species of Pacific salmon and some life-history traits [44]. Quinn's work [70] provides a meticulous summary of life history traits in an ecological context of Pacific salmon, and Hendry and Stearns provide a detailed summary of work focused on the evolution of the species [45]. We propose that Pacific salmon species provide a unique model to study the functional consequences of these amino acid substitutions because they demonstrate diverse life history types and can be easily studied in the laboratory and in the wild.

Given that the sites of protein-protein interactions identified in this study probably coordinate the translocation of H^+^ ions from the matrix to the inner membrane space, it is likely that the positively selected mutations influence the electrochemical gradient, which is comprised of both a voltage potential and a difference in pH. The magnitude of the pH gradient influences respiratory control [2] and can alter the production of reactive oxygen species, which has affects on aging among other things [5], [71], [72]. When a higher resolution structure becomes available, it should be possible to determine which of the specific amino acids in the ND5 piston arm interact with which proton pumps and the nature of the changes in the interactions that result at the sites under positive selection. This might provide information to determine if the piston arm is more tightly or loosely coupled to the pumps and therefore if pumping is made more or less efficient by the amino acid substitutions.

### Future directions

In addition to suggesting the functional and structural context that mediates the selective effects of substitutions at those seven sites, these results lead to hypotheses that can be explicitly tested in the lab. For example, a recent site-directed mutagenesis analysis of NuoL showed that mutation of a site that was detected by TreeSAAP and statistically coupled to the sites under selection (site #178) caused a significant reduction in Complex I activity [73], [74]. Other work has shown that removal of the piston arm also reduces Complex I activity [75]. Our results provide additional sites that can be tested for altered Complex I activity with mutational analyses and also provide methods to carry out a SCA in other taxonomic groups for other functionally important sites to empirically test in model organisms.

It is not possible to correlate the selected amino acid sites with Pacific salmon life-history at this point. Empirical studies that established functional differences among species would make this connection possible. The sites under selection that were detected by the codon substitution models and the TreeSAAP analysis and their position in the 3-dimensional structure suggest that they may be involved in the movements of the H^+^ ions across the inner membrane. One way to examine this hypothesis would be to isolate mitochondria from each of the eight species of salmonids and measure the oxidative output under diverse controlled conditions. From the results presented here, one might expect that mitochondria of *O. tshawytscha* would behave differently than those of the other seven species. To eliminate differences caused by nuclear-coded mitochondrial proteins of the species, one could express the different mitochondrial types in the same nuclear background in cybrid cell lines, which have been produced for numerous species [76]. These cell lines could be tested for variability of the pH gradient across the inner membrane, production of reactive oxygen species, and generation of ATP under controlled conditions.

Our SCA identified potentially important regions within the ND5 protein with respect to the sites under selection. However, this may have been simple phylogenetic signal, and we were not able to identify specific sites that were coupled to the positively selected sites with any certainty. A high-resolution structure might allow a more thorough and accurate SCA to be performed. Firstly, it would allow a structure-based alignment to be used for the MSA, which would allow a more diverse sample of taxa to be sampled for ND5, and secondly, analysis of other proteins could identify amino acid sites in other Complex I proteins that interact with the positively selected sites in ND5.

Our discovery of positive selection in a protein that is central to energy metabolism establishes an explicit connection between molecular evolution, protein function, and respiration. The final task will be to determine the physiological impacts of the ND5 and ND2 differences among species and life-history types. This study also provides a template to study the structure and function of other membrane-bound protein pumps, most of which remain unresolved.

## Supporting Information

Figure S1 **Amino acid frequency distribution at two positions (shown in shades of red) in the MSA as compared with the mean amino acid frequencies estimated from a reference set of 925,462 mitochondrially-encoded amino acid sequences of metazoans (shown in black).** (A) Frequency distribution at position 611 in both the full MSA (dark red) and the same position in a subsample obtained by randomly deleting half of the sequences (light red). (B) Frequency distribution at position 5 in both the full MSA (dark red) and the same position in a subsample obtained by randomly deleting half of the sequences (light red). Note that in the full MSA position 611 has *ΔG* score of 0.191 and position 5 has a *ΔG* scores of 0.474.
(TIF)

Figure S2 **Three phylogenetic trees constructed in MrBayes for the fifteen species from Salmonidae, which have posterior probabilities of (a)** ***PP*** **= 0.909, (b)** ***PP*** **= 0.084, and (c)** ***PP*** **= 0.007.**
(TIF)

Table S1
**The statistical energy score (** 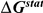 **) for eleven sites as inferred from the full alignment and from the same sites under two alternative methods of sampling from full alignment.** 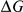-full indicates a site-specific 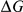 score from the full MSA. A sub-alignment was sampled from the full MSA by randomly deleting half of its sequences. The mean and SD of site-specific *ΔG* scores are given for *N* = 10 replicates of this strategy. Sequences were also sampled from the full MSA such that no divergences were permitted to be less than 5%. “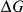-5% cutoff" denotes the site-specific 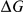 scores estimated from this method of sampling from the full MSA.(DOC)

Table S2
**The statistical energy score (** 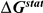 **) for seven sites as inferred from the full alignment and from the same sites after randomly sampling a subset equal to the original permutation size (** ***s*** **) for a given site.** 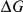-full indicates a site-specific 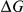 score from the full MSA. The subset size (s) is the size of the permutation of the full MSA based on the size of the most prevalent amino acid at that site. The mean and SD of site-specific 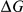 scores are given for *N* = 10 replicates where the full MSA was randomly sampled to yield *s*.(DOC)
