# Correction: Positive Darwinian Selection in the Piston That Powers Proton Pumps in Complex I of the Mitochondria of Pacific Salmon

**DOI:** 10.1371/annotation/8a7e2019-c039-4a54-b797-e9adb2ce4efc

**Published:** 2012-08-23

**Authors:** Michael R. Garvin, Joseph P. Bielawski, Anthony J. Gharrett

There were errors in the affiliations of the second and third authors. Joseph P. Bielawski's correct affiliation is 3 Department of Mathematics & Statistics, Dalhousie University, Halifax, Nova Scotia, Canada. Anthony J. Gharrett's correct affiliation is 1 Fisheries Division, School of Fisheries and Ocean Sciences, University of Alaska Fairbanks, Juneau, Alaska, United States of America. There should be no affiliation 2. 

