# Supplementary figures and images for: Positive Darwinian Selection in the Piston That Powers Proton Pumps in Complex I of the Mitochondria of Pacific Salmon
Source: PLoS One. 2011 Sep 28;6(9):e24127. doi: 10.1371/journal.pone.0024127 (PMC3182164; doi:10.1371/journal.pone.0024127)

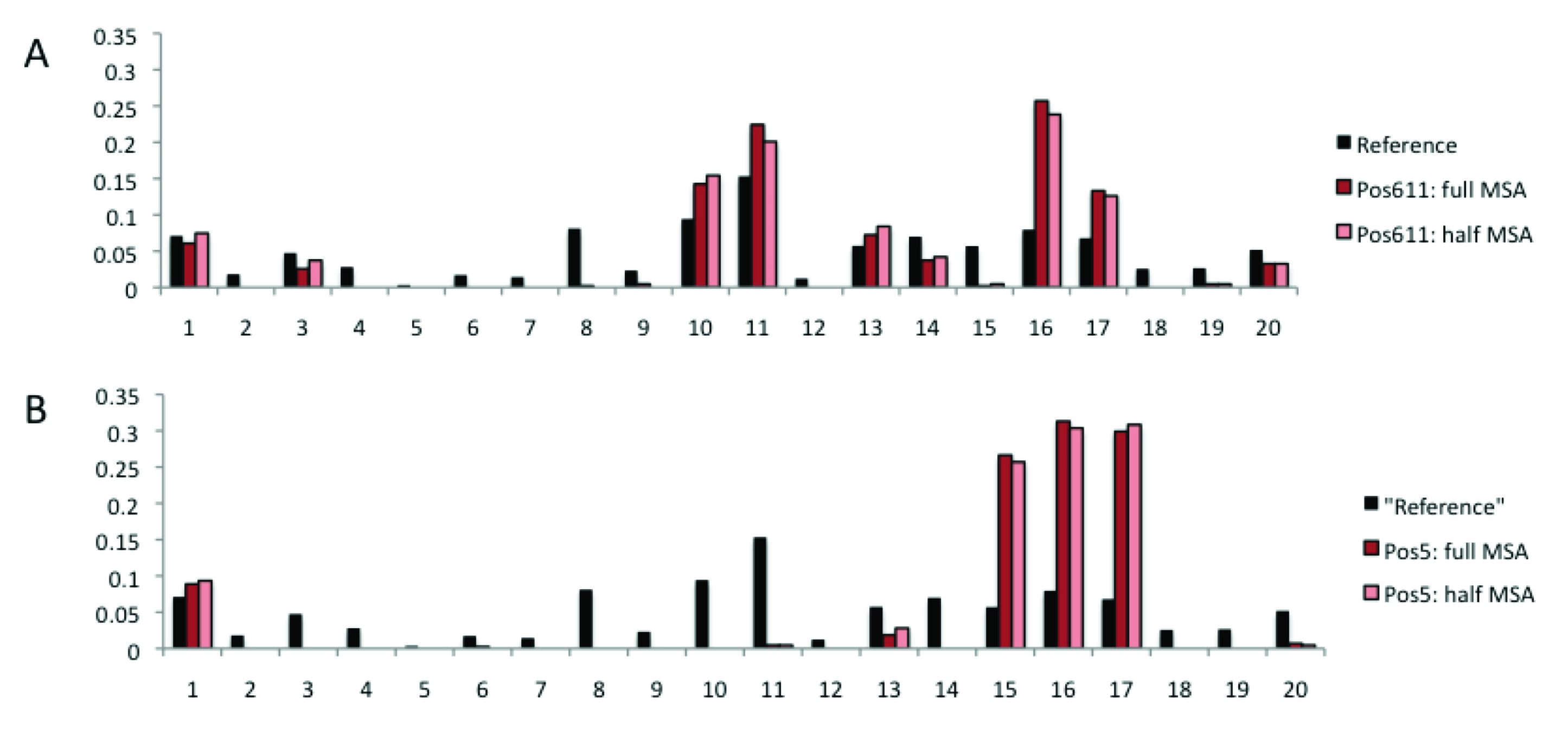

Supplement: Figure S1 — Amino acid frequency distribution at two positions (shown in shades of red) in the MSA as compared with the mean amino acid frequencies estimated from a reference set of 925,462 mitochondrially-encoded amino acid sequences of metazoans (shown in black). (A) Frequency distribution at position 611 in both the full MSA (dark red) and the same position in a subsample obtained by randomly deleting half of the sequences (light red). (B) Frequency distribution at position 5 in both the full MSA (dark red) and the same position in a subsample obtained by randomly deleting half of the sequences (light red). Note that in the full MSA position 611 has ΔG score of 0.191 and position 5 has a ΔG scores of 0.474. (TIF) [file pone.0024127.s001.tif]

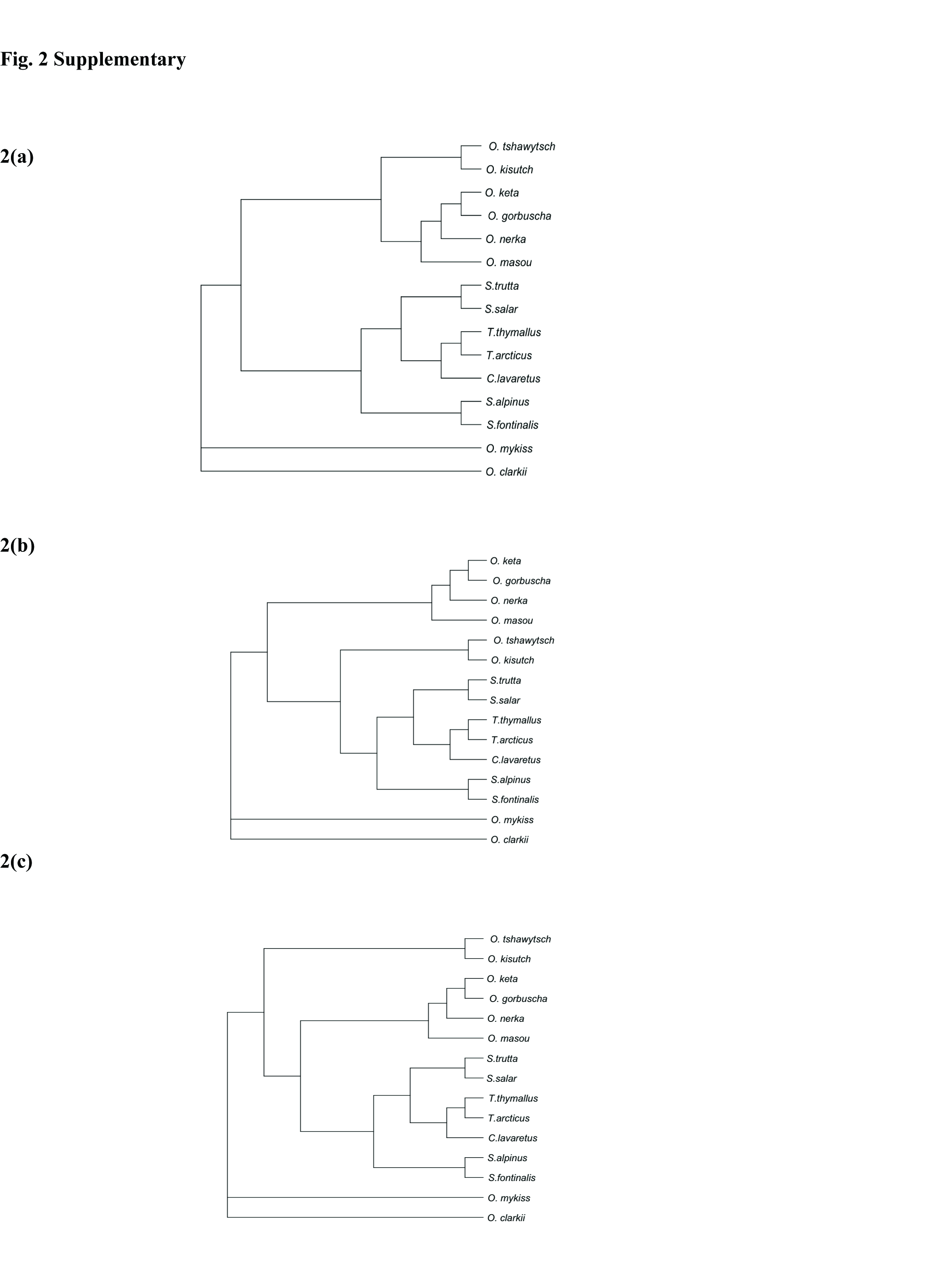

Supplement: Figure S2 — Three phylogenetic trees constructed in MrBayes for the fifteen species from Salmonidae, which have posterior probabilities of (a) PP = 0.909, (b) PP = 0.084, and (c) PP = 0.007. (TIF) [file pone.0024127.s002.tif]
