# Supplementary material for: Positive Darwinian Selection in the Piston That Powers Proton Pumps in Complex I of the Mitochondria of Pacific Salmon
Source: PLoS One. 2011 Sep 28;6(9):e24127. doi: 10.1371/journal.pone.0024127 (PMC3182164; doi:10.1371/journal.pone.0024127)
Supplement: Table S1 — The statistical energy score ( ) for eleven sites as inferred from the full alignment and from the same sites under two alternative methods of sampling from full alignment. -full indicates a site-specific score from the full MSA. A sub-alignment was sampled from the full MSA by randomly deleting half of its sequences. The mean and SD of site-specific ΔG scores are given for N = 10 replicates of this strategy. Sequences were also sampled from the full MSA such that no divergences were permitted to be less than 5%. “-5% cutoff" denotes the site-specific scores estimated from this method of sampling from the full MSA. (DOC) [file pone.0024127.s003.doc]

Table S1.

| **Random delete-half (N=10)** | | | | | | | | | |
| --- | --- | --- | --- | --- | --- | --- | --- | --- | --- |
|  |  |  |  |  |  |  |  |  |  |
|  | **Site ID** |  | ***ΔG*-full** |  | **Mean *ΔG^stat^*** |  | **SD *ΔG^stat^*** |  | ***ΔG^stat^* -5% cutoff** |
|  | 77 |  | 0.215 |  | 0.230 |  | 0.014 |  | 0.279 |
|  | 209 |  | 0.237 |  | 0.248 |  | 0.008 |  | 0.274 |
|  | 506 |  | 0.299 |  | 0.328 |  | 0.025 |  | 0.311 |
|  | 525 |  | 0.254 |  | 0.266 |  | 0.022 |  | 0.265 |
|  | 573 |  | 0.280 |  | 0.297 |  | 0.012 |  | 0.314 |
|  | 576 |  | 0.192 |  | 0.206 |  | 0.016 |  | 0.214 |
|  | 578 |  | 0.219 |  | 0.232 |  | 0.011 |  | 0.227 |
|  | 609 |  | 0.256 |  | 0.269 |  | 0.029 |  | 0.297 |
|  | 610 |  | 0.202 |  | 0.223 |  | 0.019 |  | 0.223 |
|  | 611 |  | 0.191 |  | 0.199 |  | 0.016 |  | 0.128 |
|  | 612 |  | 0.230 |  | 0.253 |  | 0.016 |  | 0.218 |
