# Supplementary material for: Positive Darwinian Selection in the Piston That Powers Proton Pumps in Complex I of the Mitochondria of Pacific Salmon
Source: PLoS One. 2011 Sep 28;6(9):e24127. doi: 10.1371/journal.pone.0024127 (PMC3182164; doi:10.1371/journal.pone.0024127)
Supplement: Table S2 — The statistical energy score ( ) for seven sites as inferred from the full alignment and from the same sites after randomly sampling a subset equal to the original permutation size ( s ) for a given site. -full indicates a site-specific score from the full MSA. The subset size (s) is the size of the permutation of the full MSA based on the size of the most prevalent amino acid at that site. The mean and SD of site-specific scores are given for N = 10 replicates where the full MSA was randomly sampled to yield s. (DOC) [file pone.0024127.s004.doc]

Table S2

| **Random sample of s (N=10)** | | | | | | | | | | |
| --- | --- | --- | --- | --- | --- | --- | --- | --- | --- | --- |
|  |  |  |  |  |  |  |  |  |  |  |
| **Site ID** |  | **Class** |  | ***ΔG*-full** |  | **Subset size *s*** |  | **Mean *ΔG^stat^*** |  | ***SD ΔG^stat^*** |
| 520 |  | E (*ω* > 1) |  | 0.709 |  | 194 (P) |  | 0.676 |  | 0.055 |
| 521 |  | E (*ω* > 1) |  | 1.759 |  | 293(T) |  | 1.917 |  | 0.026 |
| 525 |  | LT (*ω* > 1) |  | 0.254 |  | 96 (S) |  | 0.316 |  | 0.028 |
| 526 |  | LT (*ω* > 1) |  | 0.435 |  | 147 (P) |  | 0.431 |  | 0.045 |
| 575 |  | E (*ω* > 1) |  | 1.416 |  | 325 (S) |  | 1.416 |  | 0.051 |
| 576 |  | E (*ω* > 1) |  | 0.192 |  | 135 (L) |  | 0.205 |  | 0.019 |
| 577 |  | E (*ω* > 1) |  | 1.307 |  | 256 (N) |  | 1.308 |  | 0.027 |
